# Lateral ventricle central neurocytoma with lipidization: a case report of an underrecognized presentation

**DOI:** 10.1055/s-0045-1806925

**Published:** 2025-04-22

**Authors:** Daniella Karassawa Zanoni, Osorio Lopes Abath Neto, Nitesh Shekhrajka, Márcio Luís Duarte, Leonardo Furtado Freitas

**Affiliations:** 1 University of Iowa Health Care, Department of Radiology, Iowa City IA, United States.; 2 University of Iowa Health Care, Department of Pathology, Iowa City IA, United States.; 3 Universidade de Ribeirão Preto, Departamento de Radiologia, Guarujá SP, Brazil.; 4 Diagnósticos da América S.A., Departamento de Radiologia, São Paulo SP, Brazil.; 5 Baptist Health South Florida, Department of Radiology, Miami FL, United States.; 6 Radiology Associates of South Florida (RASF), Miami FL, United States.; 7 Clinical Associate Professor – Florida International University (FIU) – Herbert Wertheim College of Medicine, Miami FL, United States.

**Figure 1:**
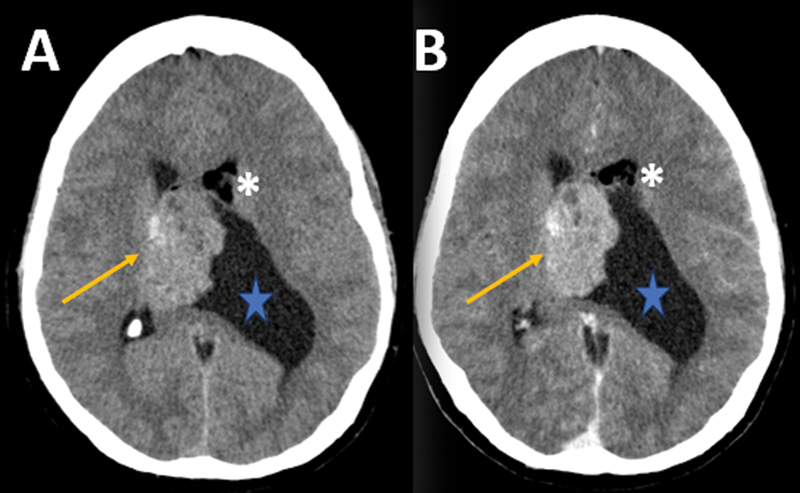
Noncontrast ( **A** ) and postcontrast ( **B** ) axial head computed tomography (CT) scans. Lobulated intraventricular solid lesion (orange arrows), predominantly on the right and centered on the septum pellucidum, with faint enhancement. Adjacent fat foci (white asterisks) demonstrated in the left frontal horn. Fluid entrapment of the left lateral ventricle (blue stars) was noted, secondary to obstruction of the left foramen of Monro (not shown).

A 38-year-old male patient with a 2-week history of intermittent somnolence, recent headaches, and blurred vision presented with a heterogeneous lateral ventricular lesion containing intralesional fat ( Figures 1 2 ). Histopathology confirmed a central neurocytoma ( Figure 3 ), a rare World Health Organization (WHO) grade 2 neuronal tumor, with lipidized cells displaying fat vacuoles but no true adipose metaplasia. Immunohistochemistry showed strong synaptophysin positivity and a low K _i_ -67 index. This case highlights the rarity of lipidization in lateral ventricular neurocytomas and emphasizes the critical role of neuroradiologists in identifying these features. The presence of fat alone does not confirm liponeurocytoma, and a detailed diagnostic correlation is required. 1 2 3

**Figure 2:**
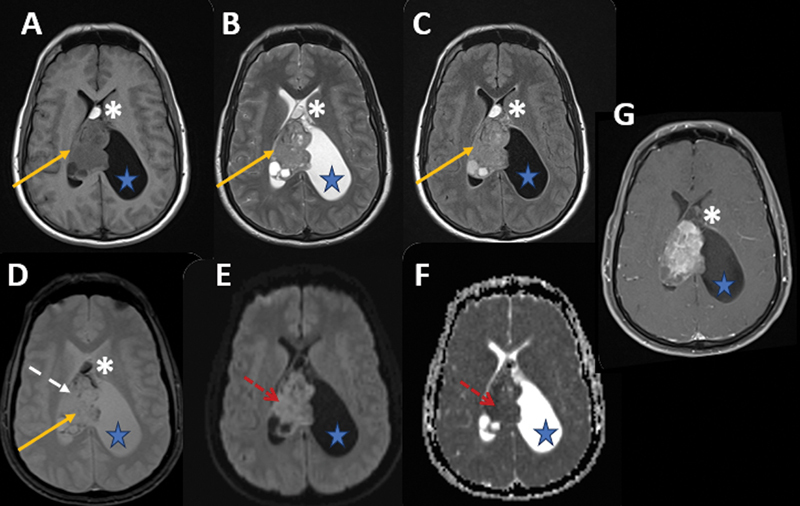
Axial brain magnetic resonance (MR) images on T1-weighted ( **A** ), T2-weighted ( **B** ), fluid-attenuated inversion recovery (FLAIR) ( **C** ), T2*-weighted ( **D** ), diffusion ( **E** ), apparent diffusion coefficient (ADC) map ( **F** ), and fat-saturation postcontrast ( **G** ) sequences. The intraventricular lobulated solid lesion (orange arrows), predominantly on the right and centered on the septum pellucidum, shows greater conspicuity, with features of microcystic degeneration, hemosiderin foci (white dashed arrow), restricted diffusion (red dashed arrows), and heterogeneous enhancement ( **G** ). A small adjacent fat focus (white asterisks) was also observed in the anterior septum pellucidum. There was fluid entrapment of the left lateral ventricle (blue stars) secondary to obstruction of the left foramen of Monro (not shown).

**Figure 3:**
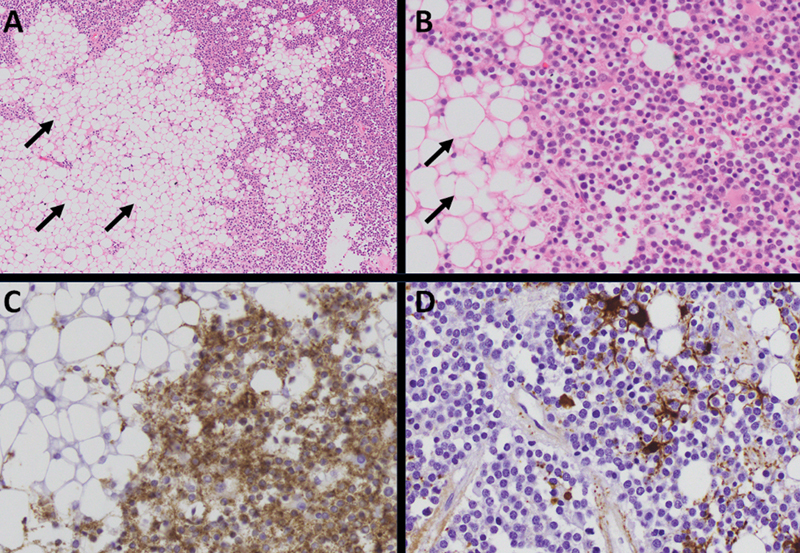
Histopathological features of the central neurocytoma. Hematoxylin and eosin (H&E) stained slides ( **A** 40X; **B** : 200X) show a neoplasm composed of monomorphic cells with round enlarged nuclei with a stippled chromatin distribution and moderated amounts of eosinophilic cytoplasm. Intimately associated with the neoplastic cells are adipocytes (black arrows). There is no mitotic activity, necrosis, or microvascular endothelial proliferation. Neoplastic cells, but not the adipocytes, are positive for synaptophysin ( **C** : 200X), and essentially negative for glial fibrillary acidic protein (GFAP) ( **D** : 200X), which decorates mainly reactive astrocytes and background gliosis in focal areas. Adipocytes are positive for S100 protein (not shown).
